# Effect of short-term exposure to Raag Bilawal of North Indian classical music on young Indian adults: a high-density electroencephalogram microstate study

**DOI:** 10.11604/pamj.2024.48.24.40977

**Published:** 2024-05-28

**Authors:** Abhisek Sahoo, Prashant Tayade, Suriya Prakash Muthukrishnan, Simran Kaur, Ratna Sharma, Madhavi Nayyar

**Affiliations:** 1 Department of Physiology, All India Institute of Medical Sciences, New Delhi, India

**Keywords:** Quantitative electroencephalogram, microstates, map topography, North Indian classical music

## Abstract

**Introduction:**

the objective of the study was to find out the microstate map topographies and their parameters generated during the resting state and during listening to North Indian classical Music Raag 'the Raag Bilawal'. It was hypothesized that in the resting state and during listening to music conditions, there would be a difference in microstate parameters i.e. mean duration, global explained variance (GEV), and time coverage.

**Methods:**

a 128-channel electroencephalogram (EEG) was recorded for 12 Indian subjects (average age 26.1+1.4 years) while resting and listening to music using the EEG microstate investigation. Investigation and comparison of the microstate parameters were the mean duration, global explained variance (GEV), and time coverage between both conditions were performed.

**Results:**

seven microstate maps were found to represent the resting state and listening to music condition, four canonical and three novel maps. No statistically significant difference was found between the two conditions for time coverage and mean duration. The statistical significance levels of the map-1, map-2, map-3, map-4, map-5, map-6, and map-7 for the mean duration were 0.4, 0.6, 0.97, 0.34, 0.32, 0.69, and 0.29 respectively; and for time coverage were 0.92, 0.92, 0.96, 0.64, 0.78, 0.38, and 0.76 respectively. Map-1, map-4, and map-7 were the three novel maps we found in our study.

**Conclusion:**

similarities regarding stability and predominance of maps with small vulnerability exist in both conditions indicating that phonological, visual, and dorsal attention networks may be activated in both resting state and listening to music condition.

## Introduction

The ancient Indian musical style known as North Indian Traditional Music (NICM), sometimes referred to as Hindustani music originated from the fusion of traditional Persian music with the vedic chant tradition [1]. Raags are the main idea of this music system; they are musical compositions that have the power to evoke certain emotions or moods. Previous research on Raags has revealed that different Raags generate different emotions [2-4]. The intrinsic qualities of music can determine the emotional quotient caused by it, major notes have been found to elicit happiness, whereas minor notes elicit sadness [5-7]. Raag Bilawal is a North Indian classical Raag with all major notes, which can induce happiness among the listeners [8,9]. Still, the neural mechanism behind the induction of emotions remains elusive. Such dynamic information can be evaluated by EEG, which has a higher temporal resolution [10]. When using the EEG, a correlation has been discovered between particular frequency bands and the emotional content in the music. Electroencephalogram spectral power measures from different areas of the cortex seem to suggest that neural networks, either local or distant, may be involved in processing of music and the communication between these networks may affect EEG in different frequency bands [11], such as an increase in alpha power in the parietal/occipital and frontal/temporal regions [12], increase in beta power in the right parietal/temporal cortex [13], enhanced gamma power in the right parietal region [14].

Topographic maps that quickly transition to a new configuration and are stable in that state, lasting between 80 and 120 ms, are referred to as EEG microstates [15-17]. The map topography analysis provides an understanding of reference-independent, temporal dynamic changes in the electric fields [18]. These states of map topography are highly reproducible and stable [19], and known as functional microstates, are postulated to constitute basic building elements for information processing, that is, the “atoms of thoughts” [16]. In a recent EEG study, big differences were seen between microstates in terms of expertise level in music with centro-parietal positive microstate top plots representing processing by expert musicians [20]. Another study looking at the emotional and cognitive reappraisal of happy music found four microstate topographies, one with central-parietal orientation. Two had bilateral occipital orientations, and one had bilateral parietal orientation [21].

Most investigations on the brain underpinnings of emotional responses to music have employed Western music as a stimulus. To the best of our knowledge, a study on microstate maps in Indian subjects in resting state and while listening to North Indian Classical Music (Raag Bilawal) using 128-channel EEG is not available in the scientific literature. From this fact, before conducting a full-fledged study in this direction, there is a need to test the feasibility of the research with different concerns, e.g. duration of listening, musical Raag, volume, etc. This led us to propose to design a new preliminary study that will experiment on a small scale as a beginning in a new direction in this initial study exposure to listening to music has been restricted to a small interval of time. Thus, the rationale for conducting this study was to find out how North Indian classical music affects the brain in terms of the microstates. The present study implies that knowing various microstates will help us classify different brain states induced due to listening to North Indian classical Raag Bilawal. Using these microstates the effects of music on the brain can be studied.

Previously conducted literature review helped to frame research questions, to set objectives, and to state the hypothesis as follows. Research questions were: 1) what are the microstates' topographies due to North Indian Classical Music Raag Bilawal; 2) what influence does North Indian Classical Music Raag Bilawal have on microstate metrics like mean duration, time coverage, and global explained variance? The objective of the present study was to find out the microstate map topographies and their parameters generated during the resting state and during listening to North Indian classical Music Raag ’the Raag Bilawal´.

It is hypothesized that in the resting state and during listening to music conditions, there would be a difference in microstate parameters i.e., mean duration, global explained variance (GEV), and time coverage amongst the microstate maps generated. To test this, we conducted a feasibility study to analyze EEG microstates in resting state and while listening to North Indian Classical Music (Raag Bilawal) in young Indian subjects and tested the hypothesis. Details of the experiment and methods in the conducted study have been covered in the methods section, the outcome is given and described in the results and discussion sections, and the finding summary has been reported in the conclusion section.

## Methods

Concerning the objective of the studies mentioned in the previous section, the study was conducted to test the hypothesis using the following methodology.

**Figure 1:**
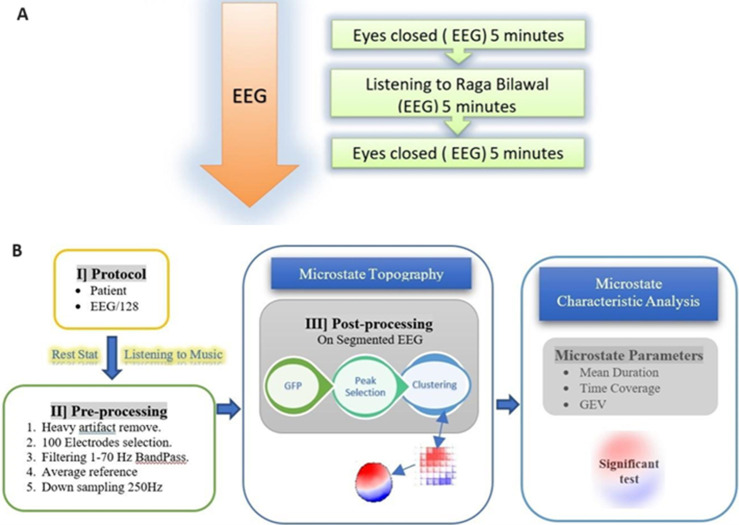
A) schematic representation of the experiment paradigm; B) outline of study design and methodology; (GEV: global explained variance, GFP: global field power)

**Study design:** the study was experimental and a feasibility (pilot) study. Following an explanation of the experimental protocol through the information sheet (in the subject's chosen language), signed informed permission was obtained from the participants. The following experimental procedure was adapted. The subject was well-rested the night before. On the day of recording after a briefing to the participant, a 128-channel HydroCel Geodesic Sensor Net (HCGSN) was placed on the scalp to record EEG. A baseline EEG recording was made with 5 minutes of eyes closed. It was followed by 5 minutes of listening to a music excerpt of 5 minutes of pre-recorded 'Raag Bilawal´ (Figure 1A). To avoid eye blinking and rolling artifacts, participants were told to close their eyes during both the resting state and during listening to the musical excerpt. The same protocol was conducted for all twelve subjects. Data was acquired and sent to conduct further work of pre-processing and further analyzed for microstates according to the adapted methodology (given in subsection 2.4).

### Experimental conduct

**Stimuli details:** North Indian music ´Bilawal Raag´ from ´Bilawal Thaat´ was selected for this work. It is a major key with a well-defined structure according to Hindustani music. The well-defined structure contained standard forward and backward progression of keys called Aaroh and Awaroh, where only ´shuddha swaras´ are used. For the present work, we reached out to professional musicians and discussed various aspects. We selected the Bilawal Raag, which usually plays in the ´first and second prahar´ of the day according to classical music. For this research, we selected the ´E´ scale and flute instrument with a background sound of ´Sitar´. Through proper standardization of instruments, Bilawal Raag was professionally recorded on the flute by an expert musician. The recorded audio was used as a stimulus to the subjects for the ´listen to music condition´ after checking that the scale of audibility was correct for each use. Stimuli were used in repeated mode during the conduct of an experiment.

**Study settings:** following recruitment, the subjects were called for recording at the stress and cognitive electro-imaging laboratory (SCEL), Department of Physiology, All India Institute of Medical Science (AIIMS), New Delhi. The recruitment of the participants was performed in December 2021. The timings of the recording were kept constant for all the subjects.

**Participants:** twelve subjects (with a mean age of 26.1 ± 1.4 years) of the 20-40-year age group of either gender who were right-handed were involved in this study. The subjects were provided with written informed consent, and all procedures (latest revision) were performed on human subjects, according to the Declaration of Helsinki for medical research, with approval from the Institutional Ethics Committee of All India Institute of Medical Sciences, New Delhi. After the assessment by Edinburgh handedness inventory (Oldfield 1971), all participants were right-handed. The participants were the institute's doctorate and postgraduate students, with no current or previous history of medical illness, psychological, substance use, or neurological issues, and no prior training in Indian classical music. The study comprised participants who had slept for at least 6.5 hours the night before. The study excluded participants who had ingested medication, alcohol, medicines, or coffee during the previous six hours.

**Variables:** investigation and comparison of the EEG microstate parameters such as mean duration, global explained variance (GEV), and time coverage between both conditions (as North Indian classical music Raga Bilawal and resting condition) were performed.

**Data sources/measurements:** the data sources and measurements include the following:

### Electroencephalogram characterization details

**Electroencephalogram data acquisition:** the study made use of a 128-electrode HCGSN system from Electrical Geodesics Inc. in Eugene, USA. Vertex (Cz) was employed as the reference electrode for the capture of the EEG data, which had a bandwidth of 0.05 to 100 Hz. The EEG was captured in a room with minimal background noise-optimized conditions. Before EEG acquisition, the impedance of each sensor was checked and found to be less than 50k Ω [19].

**Electroencephalogram pre-processing:** the EEGLAB toolbox was used to pre-process the EEG data [18]. At 1-70 Hz, a bandpass filter was used. Independent component analysis was employed using the Runica algorithm from the EEGLAB toolkit. Electrocardiography (ECG) artifacts, eye movements, eye blinks, muscle artifacts (50 Hz in India), and line noise were removed from the EEG. A total of 100 electrodes were used, with 28 eliminated from the outermost circumference due to their placement over the face and neck. Electroencephalogram data was average-referenced, and downsampling to 250 Hz was performed [19].

**Methodology for the analysis of electroencephalogram microstates:** the progression of the experiment in terms of study design and its methodology is illustrated in Figure 1B. Global field power (GFP) was used to analyze microstates. Global field power quantifies the standard deviation of geographically distributed scalp electric field potentials and GFP peaks signify functional microstates [22]. The EEG data were segmented using CARTOOL software [18]. Microstates analysis was performed on these segmented EEG epochs [16]. First, the classes of scalp template maps (topography) within each subject across both the conditions (resting state and listening to music) were discovered, which were then used in a k-means cluster analysis to produce the most predominant microstate maps [23]. To determine the number of sufficient clusters, the cross-validation criterion was used (as reported by Pascual-Marqui RD *et al*.), with the minimum value being optimal [23]. Based on GEV and mean duration, the best representative microstate maps were identified, within each subject for both the conditions (resting state and listening to music). Then, across subjects, the scalp topographies best representing both conditions were found. The maps were subjected to the k-means cluster analysis again, and the cross-validation criterion was utilized for the optimal solution. The spatial correlation between microstates in resting state and during listening to music conditions was calculated.

**Bias:** the potential sources of bias were addressed by separate EEG acquisition and pre-processing done by different investigators. Microstate analysis was done by different a investigator.

**Study size:** since it was a feasibility study convenient sampling of a sample size of 12, was done to check the impact of North Indian Classical Raga Bilawal on EEG microstate parameters.

**Quantitative variables:** the microstates were generated between two conditions using K means cluster analysis. These generated microstates were then analyzed for microstate parameters such as mean duration, time coverage, and global explained variance (GEV) between resting/baseline and North Indian Raga Bilawal condition. There was a single group that underwent pre and post-Balawal raga exposure.

**Statistical methods:** the paired t-test was used to compare the time coverage, GEV, and mean duration of all microstate maps between the two conditions. As a result, the microstate maps were differentiated between both the conditions (resting state and listening to music) based on these parameters. The differences in time coverage and mean duration and GEV (global explained variance) of microstates map between NICM and resting condition was the research question of the study to see the effect of North Indian Classical Music on Microstates.

## Results

**Participants:** twelve subjects (of a mean age of 26.1 ± 1.4 years) of the 20-40-year age group who were right-handed, and of either gender were involved in this study. All Participants participated throughout the study. The same group was exposed to raga Bilawal.

**Descriptive data:** the participants were the institute´s doctoral and postgraduate students, with no prior training in Indian classical music. No missing data as all participants were enrolled.

**Figure 2:**
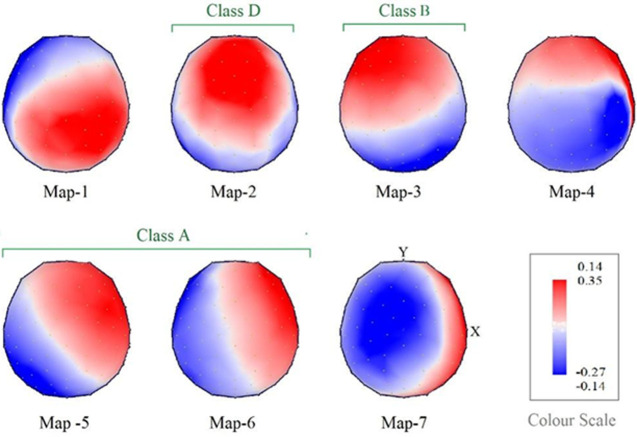
map topographies as map-1, map-2, map-3, map-4 from the left to right sequentially on top, and map-5, map-6, map-7 from the left to right sequentially on bottom

**Figure 3:**
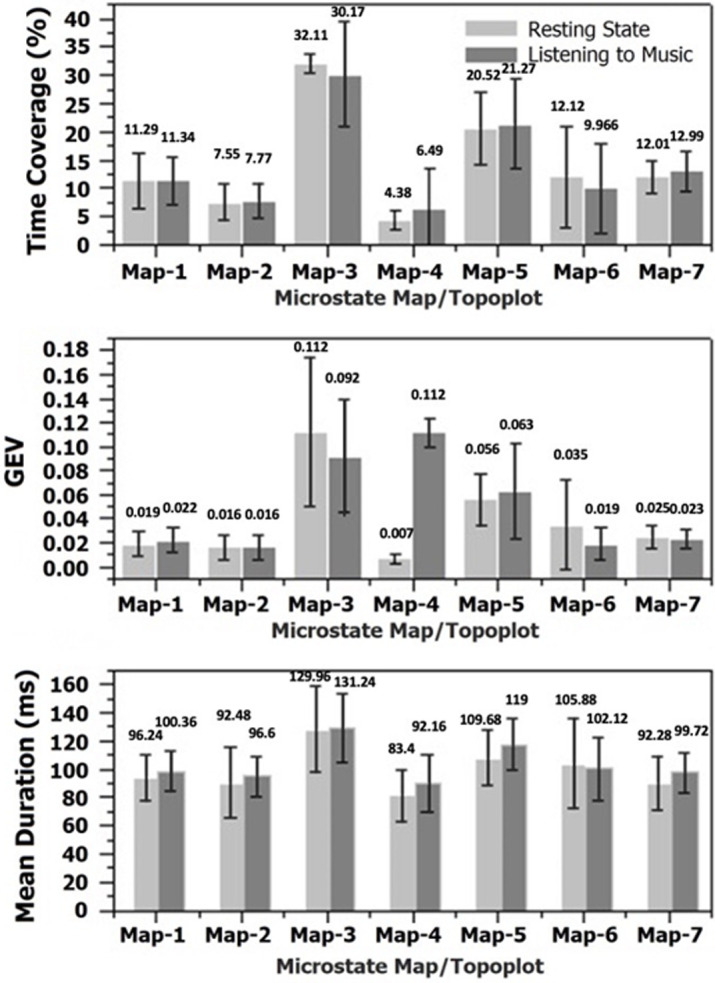
graphical representation of duration, global explained variance (GEV) and time coverage (from bottom to top) for the found microstates topo plots; (GEV: global explained variance)

**Figure 4:**
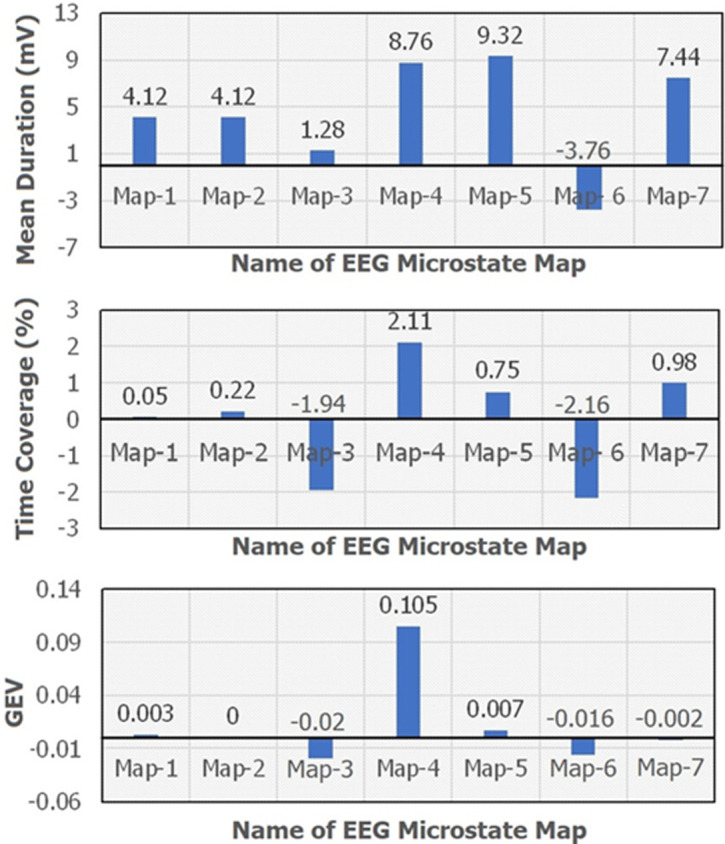
the bar plot showing the difference between the means of duration, global explained variance (GEV), and time coverage of listening to music and resting state conditions; (GEV: global explained variance)

**Outcome data:** a total of seven scalp maps of microstates out of which three map topographies were novel for Raga Bilawal. These 3 maps could be a biomarker for the effect of Raga Bilawal in inducing emotional states. The results are presented as: i) microstates map (EEG topography) between NICM and Resting conditions Figure 2; ii) microstates parameters between NICM and resting conditions. The variables used were Mean duration, GEV, and time coverage of microstate map topographies Figure 3; iii) differences between the means of microstates parameters (mean duration, time coverage, and GEV) for each map Figure 4.

**Microstates maps electroencephalogram topography perspective:** a total of seven scalp maps of microstates, sequentially map-1, map-2, map-3, map-4, map-5, map-6, and map-7, were found in the careful investigation. Their topographical illustrations (topo plots) are provided in Figure 2. These were created using k-means cluster analysis. Yellow dots on the maps represent the locations of the electrodes. The positive and the negative values of the intensity of the scalp's electrical potentials are represented by the colours red and blue, respectively, on colour scales. The positions of the spatial minima and maxima of the scalp potential are represented by the cross symbols in the colours blue and red.

**Microstates parameters outcomes:** three microstates´ parameters were obtained for map-1, map-2, map-3, map-4, map-5, map-6, and map-7 in analysis; these were mean duration, temporal coverage, and global explained variance. Details are provided in Table 1. From the tabulated result (mean), the mean duration and coverage are observed as a minimum for map-4 and maximum for map-3; the GEV is observed as the minimum for map-4 and maximum for map-3 in the case of resting state, and minimum for map-2 and maximum for map-4 in case of listening to music condition. Their overall perspective and comparison can be seen from the illustration provided in Figure 3.

**Table 1:** microstate classes and their parameters

Microstates parameters		Map-1	Map-2	Map-3	Map-4	Map-5	Map- 6	Map-7
Mean duration (ms)	RS	96.24 ±16.28	92.48 ±24.4	129.96 ±30	83.4 ±18	109.68 ±19.6	105.88 ±31.2	92.28 ±18.92
	LM	100.36 ±13.88	96.6 ±13.6	131.24 ±24.4	92.16 ±20.16	119 ±18	102.12 ±22.76	99.72 ±14.08
	PV	0.4	0.6	0.97	0.34	0.32	0.69	0.29
Time coverage (%)	RS	11.29±5.07	7.55±3.17	32.11±1.74	4.38±1.74	20.52±6.41	12.12±8.96	12.01±2.96
	LM	11.34±4.26	7.77±3.10	30.17±9.32	6.49±6.93	21.27±7.94	9.96±8.08	12.99±3.46
	PV	0.92	0.92	0.96	0.64	0.78	0.38	0.76
GEV	RS	0.019±0.01	0.016±0.01	0.112±0.062	0.007±0.004	0.056±0.021	0.035±0.037	0.025±0.01
	LM	0.022±0.01	0.016±0.01	0.092±0.047	0.112±0.012	0.063±0.039	0.019±0.013	0.023±0.008
	PV	0.54	0.97	0.52	0.83	0.41	0.35	0.77

Note: 1) RS: resting State, LM - listening to music and PV: P-value (2-tailed); 2) RS and LM values are represented in the form of (mean+SD), where SD is standard deviation, GEV: global explained variance.

The paired t-test was used to compare the time coverage, GEV, and mean duration of all microstate maps (Topo plots) between the two conditions. The result of the paired t-tests was expressed in a p-value (pairwise) row. P<0.05 was considered statistically significant. A non-significant difference was observed between the microstate parameters from the paired t-test from the p-value tabulated in Table 1, as this leads to stopping the further study. Apart from the statistically non-significant difference between microstates of resting state and listening to music intended to neglect changes, there is an observable difference between both conditions (for their mean values) which cannot be avoidable. Figure 4 is showing this difference between listening to music and resting conditions´ Means. The difference is smaller in mean values but not neglectable. The similarity of both conditions may be affected up to a smaller degree of stability and predominance of the map, however, it would be non-significant.

## Discussion

**Key results:** in an investigation to understand the EEG microstates in resting state condition and during listening to North Indian Classical Music (Raag Bilawal) in young healthy Indian participants (age 26.1±1.4 years), seven microstates´ topographies were discovered in our analysis in both circumstances. From those, four maps (topo maps) resembled classical maps of Lehmann *et al*. (1987) [16], and the rest three maps were novel and spatially distinct from the canonical maps.

**Interpretation:** each of these microstate maps representing neuronal activity has a unique hemodynamic equivalent. Many attempts have been made to correlate the microstate maps to their underlying neural networks by simultaneous functional magnetic resonance imaging (fMRI) and EEG studies. The map-2 in our study resembled class D of Koenig *et al*. [24] which has a front-to-central maximum. The Resting State Network (RSN) associated with this map has been found by Britz *et al*. (2010) to be correlated with negative BOLD activation in ventral and dorsal areas of the frontal and parietal cortices which were lateralized towards the right, representing the dorsal attention network [25]. Electroencephalogram-based Resting State Networks (eRSNs) estimation has shown that this map is associated with stronger activation of the right inferior parietal lobe (BA40) and the right mid and superior frontal gyri with activations in the right insula (BA13) [26]. It has been hypothesized that microstate D represents the switching of focus, reflexive attention, and reorientation that are more frequent during rest [27].

Map-3 resembled class B of Koenig *et al*. [24] which was correlated with negative BOLD activation in bilateral extrastriate visual cortices, broadman area 18 and broadman area 19 by Britz *et al*. representing a visual network [25]. Electroencephalogram-based Resting State Networks (eRSNs) estimation has shown that the left and right occipital cortices including the primary visual cortex (Brodmann areas 17 and 18) have shown higher activity associated with this topography with smaller areas of activation in the right insular cortex till right frontal eye field and the right claustrum (BA8). As the participants had their eyes closed, map-3 yielding visual processing would indicate visual imagery that the participants showed while listening to music and during resting state.

The other two topo plots, map-5 and map-6 resembled class A correlating with negative BOLD activation in the bilateral middle and superior temporal lobe suggesting phonological processing [25]. Electroencephalogram-based Resting State Networks (eRSNs) estimation has shown that the cortical regions associated with map-5 and map-6 topographies are the left insular cortex and left mid and superior temporal lobe i.e. Brodmann areas 22 (BA22, Wernicke area), and area 41 (BA41, primary auditory cortex) and with lower activation in the left lingual gyrus (BA19) [26]. Listening to music recruits neural repertoire for decoding the auditory information, but the presence of the map during the resting state suggests that these networks might also be active during the resting state. These results are indicating that the phonological and visual processing with attentional networks might be activated in both conditions of listening to music and resting state. Apart from these, there were 3 novel maps map-1, map-4, and map-7 which to the best of our knowledge have not been described in previous studies. These microstates' topographies could be the markers of the emotional state generated by listening to Raag Bilawal. Raag Bilawal with all major notes elicits emotions with positive valence [9]. It is a soothing and happiness-inducing Raag that would lead to a mental state of generation of positive emotions.

According to the literature, the mean duration of a microstate is the average amount of time, the microstate remains stable [16] and the temporal coverage [16] means the proportion of total time, a microstate map remains dominant. Moreover, the global explained variance or GEV of microstates refers to the overall variance explained by a certain microstate in percentage which is the map's ability to describe the strength with which it displays data as well as its frequency of occurrence [26]. Thus, map-3 seems to be the most stable and dominated for the longest time coverage, and the map-4 is least stable and has the smallest dominant coverage.

It was hypothesized that in the resting state and during listening to music conditions, there would be a difference in microstate parameters. However, from the p-value, there was no significant difference observed in the maps between the resting state and during listening to music condition for all parameters including mean duration, GEV, and time coverage. This suggests that the two conditions were similar in terms of stability and predominance of maps. However, there were observable differences in the mean values of these parameters between the two conditions mentioned in Figure 4. While the microstates during listening to music and resting state indicate simultaneous increased phonological processing and visual processing with dorsal attention network activation, there could be different networks activated as well. Hence, further exploration of large data might yield a clearer understanding of the microstates of listening to music.

On the analysis though there was no significant difference, but there was an observational trend shown in Figure 4, the mean duration is positive for all maps except map-6; map-6 shows a negative difference and indicates the decrement of temporal duration for the corresponding microstate. It can be represented as the phonological process may be vulnerable (or perturbing) to getting decreased. Similarly, coverage is decreasing by a percentage of 1.94 and 2.96 for map-3 and map-6. Map-3 and map-6 topographies are also negative in GEV. It represents a vulnerability (perturbation) to decrement in salient network and phonological processes. Map-4 is a novel topo plot and its all-microstate parameters show consistent and good characteristics of positive difference. This novel map-4 needs to be characterized in further studies and could serve as a biomarker for the underlying neural mechanisms as an effect of listening to North Indian Classical music.

**Limitations and future implications:** this work has a huge scope in the future subjected to cross the present limitations: (i) the present study was done with a small sample size; further research on a larger sample size could generate substantial findings; (ii) the source analysis and functional connectivity analysis of the acquired microstates would reveal distinct brain regions and network connectivity between the areas for the microstates, which must be investigated further; (iii) short-term exposure to listening to music stimulus did not work as per our hypothesis; longer time exposer study design of Raag Bilawal could be tested further.

**Generalizability:** this study was performed on Indian participants and the three novel maps found in this study could reflect the changes induced by Raga Bilawal in a large population if explored for a long duration. These map topographies could be generalizable to the young Indian population.

## Conclusion

Electroencephalogram microstates while listening short time exposure to Raag Bilawal in young healthy Indian individuals were successfully studied by taking all precautionary measures. The study has reported that: (i) 7 microstate maps were found to represent the resting state and listening to music condition; (ii) statistically, there was no significant difference found between the two conditions for the parameters such as time coverage and mean duration. It indicates that both conditions must be comparable in terms of stability and predominance of maps. As revealed by various findings from different studies, networks such as phonological, visual, and dorsal attention networks may be activated in both resting state and listening to music conditions, reflecting these seven microstates, which need to be investigated further in listening to music conditions.

### What is known about this topic

- *Music can induce emotions among the listeners; Raag Bilawal, a North Indian classical music Raag with major notes is a soothing and happiness-inducing musical piece leading to a mental state of positive emotions*.

### What this study adds

- *This is the only study exploring 128-channel quantitative EEG microstate parameters while listening to music*;
- *Seven map topographies were obtained, out of which four were canonical and three were novel maps; these three novel maps could be the markers of the emotional state while listening to Raag Bilawal*;
- *Areas responsible for phonological and visual processing along with attentional networks might be activated during listening to music as well as during the resting state*.
